# Acceptability and Usability of Telemedicine for Older Veterans in a Pilot Sample: Mixed Methods Study

**DOI:** 10.2196/86944

**Published:** 2026-08-06

**Authors:** Mollie A Ruben, Atticus Carroll, Maria D Venegas, Chelsea E Hawley, Megan B McCullough, William W Hung, Lauren R Moo

**Affiliations:** 1 Department of Psychology, University of Rhode Island, 142 Flagg Road, Kingston, RI, 02881, United States, 1 401-874-4560; 2 Geriatric Research Education and Clinical CenterBedford, MA, United States; 3 Center for Health Optimization and Implementation Research, Edith Nourse Rogers Memorial Veterans Hospital, Bedford, MA, United States; 4 Department of Medicine, Aram V. Chobanian & Edward Avedisian School of Medicine, Boston University, Boston, MA, United States; 5 James J. Peters VA Medical CenterBronx, NY, United States; 6 Department of Geriatrics and Palliative Medicine, Icahn School of Medicine at Mount Sinai, New York, NY, United States

**Keywords:** telehealth, older adults, medication management, technology use, behavioral observation

## Abstract

**Background:**

Telemedicine offers promising solutions for improving access to care among older adults with chronic conditions, but there is limited evidence on how older patients navigate and engage with video telehealth in their home environments.

**Objective:**

This study aimed to assess older adults’ acceptability, usability, comfort, and engagement with pharmacist-led telemedicine visits conducted at home using behavioral observation separate from the telehealth platform.

**Methods:**

This descriptive mixed methods pilot study included a convenience sample of 20 older adult veterans (aged ≥65 years, ≥2 chronic conditions, and ≥5 medications) recruited from a Veterans Affairs (VA) health care system. A project manager observed and video-recorded participant preparation for and conduct during in-home telemedicine visits with a clinical pharmacist. Recordings were coded using structured protocols to assess verbal and nonverbal behavior, technological challenges, and environmental factors. Trained and reliable coders rated verbal and nonverbal comfort, frustration, and engagement during technology setup and the telemedicine appointment using Likert-type impression scales (intercoder reliability was good, Cohen κ=0.81). Video data were also transcribed and coded for behavioral events (eg, troubleshooting, verbal expressions of age or technology ability, and nonverbal adaptation) and analyzed using rapid qualitative analysis.

**Results:**

Participants expressed moderate engagement and comfort with technology overall. Verbal and nonverbal engagement significantly increased from setup to appointment (*Z*=2.03, *P*=.002). Technology-related challenges (eg, audio lag and troubleshooting) occurred in over half the visits but were often resolved through participant adaptation or support from the research staff. Participants displayed both frustration (eg, sighing and leaning away) and adaptability (eg, propping up tablets and retrieving medications). Verbal expressions reflected a mix of technology confidence and age-related limitations. Environmental distractions were present in some visits (eg, dogs barking or phones ringing), but also allowed for rich clinical engagement (eg, home tours and direct observation of medications).

**Conclusions:**

In this small pilot sample of older veterans, participants generally remained engaged during in-home telemedicine visits despite technological and contextual barriers, suggesting that home-based telemedicine may be acceptable for some older adults when support is available. Addressing technical support, home environment considerations, and age-related perceptions of technology may increase telehealth usability and satisfaction among aging populations.

## Introduction

Medications are one of the primary interventions in the treatment and prevention of disease. Older adults with chronic diseases face the difficulty of managing medication regimens and polypharmacy, as well as the challenges of mobility and transportation to get to care [1,2]. Lack of access to health care is a system-level issue affecting health care organizations, including the Veterans Affairs (VA), the largest integrated health care system in the United States. There are paths to ameliorating medication-related health care challenges for older patients. The first is using clinical pharmacy practitioners (CPPs) to deliver patient-centered comprehensive medication reconciliation in which all a patient’s medications—prescription, nonprescription, alternative, traditional, vitamins, or nutritional supplements—are assessed to establish whether the medications are safe, effective, and can be taken as intended. The second is geriatric telemedicine, which seeks to address access issues [3]. However, there remains an important gap in our understanding, as older adults are rarely included in studies on telemedicine [4], even though telemedicine eliminates the need for transportation, thereby reducing logistical barriers, allowing for more effective medication management, and potentially improving adherence to medication regimens [5].

A home video appointment format for medication management allows individuals to show the label on a bottle and how medications are stored directly to the provider. This allows the provider to make a more accurate assessment of the impacts of prescribed medications and appropriate storage [6]. Additionally, conducting appointments in the home environment gives patients an opportunity to engage with the provider in a more comfortable environment, affording the provider a multidimensional picture of their cognitive and emotional state, something not as easily attained in a clinical setting [6].

However, certain barriers may limit the widespread adoption and success of CPP-delivered telemedicine among older adults. Access to the necessary technology, such as smartphones, tablets, or stable internet connections, may be constrained due to financial, geographic, or infrastructural limitations [7]. Furthermore, older adults may hold varying levels of technological literacy, influenced by generational differences in familiarity with digital tools [8]. Beliefs about age and capability may also affect confidence and willingness to engage with technology [9]. When technological issues arise, such as connectivity problems or user interface difficulties, older adults may experience discomfort or frustration, particularly if they lack the skills or resources to troubleshoot effectively [10]. Distractions may exist in the home that do not exist in the office and may hamper the effectiveness or acceptability of telemedicine appointments.

Although prior telemedicine research has often relied on patient-reported acceptability, satisfaction, or usability measures, self-report alone may not capture how older adults actually navigate video visits in real time. Behavioral observation provides complementary and unique insight by documenting verbal and nonverbal indicators of comfort, frustration, engagement, adaptation, troubleshooting, and environmental context as they occur during the telemedicine encounter. Thus, an important knowledge gap remains regarding how older adults behaviorally engage with pharmacist-led telemedicine in the home setting, including how they respond to technology challenges and how the home environment functions as both a barrier and clinical resource.

Therefore, to reach older adults effectively with technology and carry out effective virtual medication reconciliation, we conducted a mixed methods pilot study using a convergent parallel design [11]. The convergent parallel design allowed us to simultaneously yet independently capture quantitative and qualitative data. As part of this assessment, we had the rich opportunity to capture observational data in the home, separate from the telehealth platform, to record the full context of telemedicine appointments. Observational methods are an innovative method that gets researchers very close to the actual televisit encounter between the CPP and the patient. This particular method has changed how researchers see and understand verbal and nonverbal communication in several critical areas, including family medicine [12], interactions with patients living with HIV [13], and psychiatry appointments [14], to name a few. Therefore, in this study, we coded quantitative behaviors relevant to comfort and engagement with technology and used observational methods including rapid qualitative analysis to contextualize the present work. This allowed us to characterize the engagement of patients with this telemedicine medication reconciliation process, adding deep insight into the intervention. Therefore, the purpose of this descriptive mixed methods pilot study was to characterize older veterans’ acceptability, usability, comfort, and engagement during pharmacist-led telemedicine visits conducted in the home, and to identify contextual barriers and facilitators that may inform future telemedicine design.

## Methods

### Participants

A convenience sample of 20 older adult veterans (19 men and 1 woman) from the Edith Nourse Rogers Memorial Veterans Hospital in Bedford, Massachusetts, participated in this study of the acceptability and usability of pharmacist-led telemedicine visits. Eligible participants were required to be aged 65 years or older, have attended at least 1 VA primary care visit within the prior year, have at least 2 chronic conditions, and be prescribed at least 5 daily medications. Veterans were excluded if they had a diagnosis of dementia or lacked capacity to provide informed consent. During each visit, a VA-based clinical pharmacy practitioner conducted the telemedicine appointment by video while the participant remained at home, accompanied by an in-home project manager who observed the visit and provided support when needed.

Potential participants were identified through an electronic health record–generated list of veterans who met preliminary eligibility criteria. Following a HIPAA (Health Insurance Portability and Accountability Act) waiver, approximately 3000 records were screened. Recruitment took place more than approximately 6‐10 months using mailed invitation letters followed by outbound telephone calls. A total of 316 invitation letters were mailed; 64 veterans responded, of whom 41 declined participation and 23 initially agreed. Two of those 23 later declined enrollment because of personal circumstances. To expand recruitment, study staff telephoned veterans who did not respond to the mailing. Among veterans reached by phone, 81 declined participation and 43 did not respond to contact attempts. An additional 17 veterans were found ineligible after further screening. Veterans who expressed interest were provided with study information using an institutional review board (IRB)–approved script, and those who reported not receiving the invitation letter were sent another copy upon request. Recruitment ended once the target sample of 20 participants was enrolled.

The invited recruitment pool was also limited in racial and ethnic diversity. Among the 316 veterans who were mailed invitation letters, 298 were non-Hispanic White, 1 was Hispanic White, 1 was Alaskan Native, 7 were African American, 2 were Asian, and 6 did not report race or ethnicity. The broader VA Bedford Healthcare System population of eligible veterans older than 65 years of age was also predominantly non-Hispanic White, although the invited pool included a higher proportion of White veterans and a lower proportion of Black veterans than the broader eligible population. The enrolled sample included 19 non-Hispanic White participants and 1 Asian participant, reflecting a racial composition similar to the invited pool.

### Ethical Considerations

The study was approved by the VA Central Institutional Review Board (CIRB; IRBNet ID 1612635). Before enrollment, eligible patients were assessed for capacity to provide informed consent using a screening questionnaire evaluating understanding, appreciation, ability to communicate a choice, and rationale for that choice. Written informed consent was obtained from all participants, and recruitment and consent procedures were conducted in a setting that ensured participant privacy and convenience.

To protect participant privacy and confidentiality, study data were handled by the research team for analysis purposes; findings are reported in aggregate form, and only deidentified quotations are presented in the paper. Supporting data are not publicly available because participants did not provide written consent for public data sharing. Participants received a US $50 gift card for participation.

### Procedure

This pilot study evaluated a pharmacist-led telemedicine intervention for medication reconciliation and management conducted in participants’ homes using the VA’s synchronous video platform, VA Video Connect. This study was designed as an evaluation of the telemedicine visit process in the context of study-supported implementation, rather than as an evaluation of usual care alone. Prior to the visit, study personnel ensured that participants had access to an internet-capable device with a camera and microphone or arranged for a VA-loaned tablet when needed. Participants receiving a VA-loaned tablet completed a test visit, while other participants were given a demonstration prior to the pharmacist visit.

An experienced anthropologist (MDV) serving as project manager recruited and enrolled participants, administered baseline questionnaires, and joined each participant in person at their home before and during their virtual pharmacist visit. The project manager set up study videorecording equipment that recorded the entire audio and video interaction from prior to participant technology setup through to the end of the pharmacist-patient telemedicine visit. The same project manager conducted direct observations in participants’ homes and documented both behavioral interactions and contextual barriers through structured fieldnotes.

### Technology Setup

To join a video visit, participants needed home internet access, an internet-compatible device with a camera and microphone, and access to email to open an autogenerated visit link. Participants used either their own device (eg, laptop, desktop, or tablet) or a VA-issued tablet. After opening the link, they entered their name and connected to the visit. An in-home study team member observed the setup process and, when technical problems occurred before the pharmacist joined, first gave participants an opportunity to resolve them independently before providing assistance as needed. After the participant connected, an automated message played until the pharmacist was notified and joined the visit.

### Telehealth Appointment

Participants were told the purpose of the video visit was to conduct a comprehensive medication reconciliation and management review, including visualization of medications and how they were organized in the home. After joining the visit, the pharmacist confirmed the participant’s location in case of emergency, requested a phone number in case of disconnection, and obtained permission to discuss medications. The pharmacist then completed a brief medication-focused functional assessment by asking participants to read aloud and interpret one or more prescription bottles and demonstrate the ability to open them. Medications currently being taken were reviewed and compared with the electronic medical record to identify discrepancies. To facilitate this review, participants either brought the device to where medications were stored, brought their medications to the device, or read from an up-to-date medication list. The pharmacist also provided education about possible side effects or interactions, clarified correct medication use when discrepancies were identified, and answered participant questions. After the visit, the pharmacist documented the review and any discrepancies in the electronic medical record for the primary care team.

In summary, the study timeline included recruitment and consent, baseline assessment, previsit technology preparation, in-home observation of visit setup, pharmacist-led telemedicine medication reconciliation, and postvisit coding and analysis of recorded encounters.

### Measures

#### Previous Technology Experience

Participants self-reported their previous technology experience as some experience (eg, owning and using a smartphone, tablet, or computer) or no experience (eg, no access to a smartphone or any device such as a computer desktop, laptop, or tablet).

#### Sociodemographic Information

Participants completed items about their age, sex, race, and ethnicity.

#### Observational Audio and Video Coding

We conducted direct observation by coding the video-recorded telehealth visits to systematically capture both verbal and nonverbal behaviors relevant to technology use. Coders were trained to identify, transcribe, and rate specific verbal content (eg, comments about age or knowledge about technology) and identify and code nonverbal behaviors (eg, sighs, posture, and facial expressions related to technology use) using a structured coding scheme. Coding was done at the utterance level for verbal content (ie, each complete thought or sentence) and behavioral events for nonverbal features. This approach allows for nuanced assessment of telemedicine dynamics beyond what is captured in transcripts or self-report alone.

We adapted several coding protocols used in our prior research [15,16] and Williams et al [17] telemonitoring technology use for older adults to evaluate potential issues and use of telehealth for older adult participants in their homes. Coders independently watched the video recordings and rated participants on several quantitative measures as well as described these quantitative codes qualitatively through both what the participant said (using direct quotes of their verbal behavior) and how they behaved (describing their nonverbal behavior). We captured quantitative frequency counts and qualitative descriptions of participants’ nonverbal behavior relevant to technology use, verbally expressing age or knowledge, what occurred during any troubleshooting and time spent doing so, technology interruptions, and any distraction or remark about one’s home or space. Refer to Table 1 for definitions of these codes and Multimedia Appendix 1 for the coding template.

**Table 1.** Definitions of technology codes.

Code	Definition
Nonverbal behavior relevant to technology use	Note any nonverbal behavior relevant to technology use, including sighs, movement toward or away from tech, and so on
Verbally expresses age or knowledge	Participant vocally expressed that they were “too old to learn new things” or similar
Time spent troubleshooting	Time how long it takes to start appointment
Technology interruptions	Note any interruptions in technology once appointment starts
Remark about home, space, or self	Participant or pharmacist made comment about or was distracted by home environment, space, sounds, self, including comments about going into the other room to get medication

In addition to the frequency and type of code described above, we quantitatively rated participants on 4 gestalt impression ratings prior to the pharmacist joining the video visit to examine how participants handled the technology setup in terms of their comfort, frustration, and engagement both verbally and nonverbally. We also rated these same 4 gestalt impression ratings for the duration of the telemedicine appointment, from the moment the pharmacist joined until the end of the video visit, to assess comfort, frustration, and engagement verbally and nonverbally during the actual telemedicine appointment and assess for change from the technology setup to the telemedicine appointment. Coders were instructed not to focus on specific behaviors when rating these impressions; rather, they were trained to make a holistic judgment. Refer to Table 2 for Likert-type rating scales used for each of these impression ratings. For example, a participant who leaned toward the device, maintained attention to the pharmacist, retrieved medications to show on camera, and repositioned the tablet to participate more fully would be rated as highly engaged. In contrast, a participant who looked away from the device, delayed responding to technology prompts, or relied primarily on the project manager to proceed would receive a lower engagement rating. Similarly, comfort and frustration ratings were based on both verbal and nonverbal indicators: a participant who calmly navigated the video link or expressed ease with the device would be rated as more comfortable, whereas sighing, leaning away from the device, displaying tense facial expressions, hesitating before touching the screen, or verbally expressing difficulty with the technology would contribute to higher perceived discomfort or frustration. Reliability was assessed by 1 primary coder and 2 secondary coders on 20% of the interactions. There was strong agreement between the coders’ judgments (Cohen κ=0.82, *P*<.001).

**Table 2.** Definitions of nonverbal and verbal comfort, frustration, and engagement codes rated at 2 time points, during technology setup and again during the actual telemedicine appointment.

Impression	Rating scale
Verbally expresses comfort with technology	1 (expresses extreme discomfort) 2 (expresses moderate discomfort) 3 (neither expresses discomfort nor comfort) 4 (expresses moderate comfort) 5 (expresses extreme comfort)
Verbally or vocally expresses frustration versus ease with technology	1 (expresses extreme frustration) 2 (expresses moderate frustration) 3 (neither expresses frustration nor ease) 4 (expresses moderate ease) 5 (expresses extreme ease)
Nonverbally expresses comfort with technology	1 (expresses extreme discomfort including distancing self from technology) 2 (expresses moderate discomfort) 3 (neither expresses discomfort nor comfort) 4 (expresses moderate comfort) 5 (expresses extreme comfort)
Verbal and nonverbal engagement with technology	1 (expresses extreme disengagement) 2 (expresses moderate disengagement) 3 (neither expresses disengagement nor engagement) 4 (expresses moderate engagement) 5 (expresses extreme engagement)

### Positionality

An experienced anthropologist serving as project manager (MDV) conducted in-home observations and was present during telemedicine visits, which may have influenced participant behavior, including help-seeking, self-presentation, and engagement with the technology. Coders—2 research psychologists, an anthropologist, and a cognitive behavioral neurologist—were trained using a structured coding protocol, and coding decisions were discussed across team members to support consistency. At the same time, the interpretation of verbal and nonverbal behavior was shaped by the disciplinary backgrounds and analytic perspectives of the research team, which should be considered when interpreting the findings.

### Analysis

Quantitative frequency counts and impression ratings were analyzed with means and SDs. We compared changes in impression ratings from technology setup to telemedicine appointment with a Wilcoxon signed-rank test given the nonnormal distribution of data.

To analyze qualitative data, rapid qualitative analysis was used [18]. These methods have been found to have a high level of concordance with traditional qualitative methods and are known for their utility in action-oriented, time-sensitive work [18,19]. We used a method developed by Hamilton and other researchers [18,20]. Team members created a template focused on the codes addressed in the modified coding system described above. This template was used to summarize each interaction. Three team members (MAR, MDV, and LRM) summarized the first 4 video visits and discussed findings to ensure consistency and completeness and then edited the template before summarizing the rest of the video visits. Then, a summary was completed for each of the remaining video visits by a single team member, with discussion when needed. The summaries were then combined to create a matrix of codes or domains across all 20 video visits [21]. From this matrix, the codes were reviewed and synthesized into resulting content areas, themes, and representative quotations by MR and AC.

## Results

### Sample Characteristics and Technology Experience

Participant demographic information is summarized in Table 3. Participants were on average 74 years old; most were non-Hispanic White, male, and averaged 11 prescribed medications. Of the 20 participants, 13 (65%) reported some level of experience with technology (eg, owning and using a smartphone, tablet, or computer) but for the remaining participants, access to and experience with videoconferencing apps and devices was limited or assistance was needed (eg, no access to a smartphone or any device such as a computer desktop, laptop, or tablet). The 7 (35%) participants who neither had access to a device nor experience with technology all needed assistance before and during the visit. Finally, 3 (15%) participants who had access to a device and reported some experience with technology still ran into technical issues and needed assistance before and/or during the visit.

**Table 3.** Sociodemographic information of participants, device used for telemedicine appointment, and total medications (N=20).

Sociodemographic variable	Value
Age (years), mean (SD)	74.00 (3.26)
Race, n (%)	
White	19 (95)
Asian	1 (5)
Sex assigned at birth	
Male	19 (95)
Female	1 (5)
Device used, n (%)	
Personal laptop	9 (45)
Personal tablet	3 (15)
VA^a^ tablet	8 (40)
Total medications, mean (SD)	11.25 (4.37)
Technology use experience, n (%)	
Some previous experience	13 (65)
No previous experience	7 (35)

a VA: Veterans Affairs.

### Appointment Length and Time to Start the Appointment

It took, on average, 9 minutes and 4.8 seconds (SD 9 minutes 53 seconds) for the telemedicine appointment to start from the beginning of the video recording (range: 1 minute 6.6 seconds to 43 minutes 21.6 seconds). In one case, the participant was already on the telemedicine appointment when the recording began, and they were excluded from this analysis. In another case, it took a participant 43 minutes and 21.6 seconds to gain access to the telemedicine system and for the appointment to begin because of an outdated system that wouldn’t allow access to their webcam. After troubleshooting with both the project manager in the home and the pharmacist on the video connect, this participant was successfully able to access their telemedicine appointment.

### Ratings of Comfort, Frustration, and Engagement During Technology Setup

During technology setup, participants on average were neutral regarding comfort and frustration with technology in their facial expressions and body language (nonverbal behavior) and with their verbal and vocal comments. Finally, participants displayed moderate engagement on average during technology setup (Table 4).

**Table 4.** Impression ratings of verbal and nonverbal comfort, frustration, and engagement during technology setup and during telemedicine appointments and difference from setup to appointment^a^.

Impression rating	Setup, median (IQR)	Appointment, median (IQR)	Wilcoxon signed-rank test (Z statistic)	*P* value
Verbally expresses comfort with technology	4 (2-5)	3 (2-4)	1.22	.22
Verbally or vocally expresses frustration versus ease with technology	3 (2.25‐4)	3 (2.25‐3.75)	0.24	.81
Nonverbally expresses comfort with technology	4 (2‐4.75)	4 (3-5)	1.81	.07
Verbal and nonverbal engagement with technology	4 (4-5)	5 (4.25‐5)	2.03	.002

a Rating scale ranged from 1 to 5.

### Ratings of Comfort, Frustration, and Engagement During Telemedicine Appointment

During the actual telemedicine appointment, participants on average were similarly neutral regarding comfort and frustration with technology in their facial expressions and body language (nonverbal behavior) and with their verbal and vocal comments. Participants did, on average, appear moderately comfortable with technology through their nonverbal behavior. Finally, participants appeared, on average, extremely engaged during the telemedicine appointment (Table 4). Verbal and nonverbal engagement with technology was the only impression rating to significantly change (ie, increased) from setup to appointment.

### Frequency Counts and Rapid Qualitative Analysis

Qualitative results are presented by themes within the content areas. A summary of themes within these areas is presented below and representative quotes are presented in Table 5.

**Table 5.** Quotations from and descriptions of participants regarding technology use during telemedicine appointments.

Themes within content areas	Quotations and Description
Nonverbal behavior relevant to technology use	
Nonverbal frustration	Sighing deeply, negative affective facial expressions (eg, overwhelm, distress, and irritation)
Adapting environment to technology	Figuring out a way to prop up a device to be hands-free, such as stacking books or using things already on the table that are nearby
Interpersonal distance	Leaning toward and/or moving chair toward device when engaged versus leaning away or moving chair away when frustrated or annoyed with technology
Physical hesitancy with device	Not immediately typing on the device when given prompts indicating confusion, typing slowly to not make mistakes, tentatively accepting the device from the project manager
Expressive gestures	Becoming more physically animated, such as using hands while talking when engaged in conversation
Verbally expresses age or knowledge	
Finding it difficult to keep up with technology due to one’s age	“I’m too old for this.”
Aging and ailments	“If you speak up a little bit louder or if I could get some more volume, that would be better… [turns up volume] I have hearing aids on and I hear you okay now.”
Memory loss	“I have to write all the passwords”
Limited expertise with technology	“I don’t even use it [tablet]. Not for this. I know how to make phone calls. That’s it.”
Technologically savvy or ease of accessing video link	“I am very computer savvy, so I don’t have a problem getting on and, you know, listing what I need.” “I already have it [video link] up”
Reliance on younger generations	“You know who I learned that from? My granddaughter. She knows how to do this”
Pride in aging	“How many 74-year-olds do you know who can ride 25 miles on a bike?”
Troubleshooting	
Patient problem-solving	Figured out how to flip camera on tablet Asked pharmacist to pause between question and answer because of lag
External support	The project manager closed a pop-up after the participant couldn’t figure it out on their own The project manager turned volume up after participant couldn’t figure out on own Nonverbal glances or gestures from patient to the project manager to validate or elicit support
Verbalizing next steps for validation from research assistant	“Click start?” “Want me to click and join?”
Device-specific issues	Home Wi-Fi connectivity issues Issues with laptop camera
Technology interruptions	Video froze momentarily Video was lost while the audio kept working Lag between the audio and video
Remark about home, space, or self	
Retrieve medication	Participants frequently got up to retrieve medication from another room to answer a question about dose or type
Distraction	Dogs barking, partner interrupted, or phone ringing
Home tour	Showed pharmacist around home using tablet
Self-awareness heightened	Made references to bad hair day or started fixing hair and asked if red in the face

### Nonverbal Behavior Relevant to Technology Use

Nonverbal behaviors related to technology use occurred on average 8.85 (SD 5.82) times per video visit. These behaviors ranged from signs of frustration or engagement through gesturing or reducing the interpersonal distance between the patient and the screen. A list of these codes is located in Table 5. Nonverbal frustration occurred in 5 of the video visits, mostly at the beginning when there were issues with technology and/or connecting to the appointment. This most often involved participants sighing deeply or displaying a facial expression of frustration to someone else in the room (eg, a partner). Interpersonal distance decreased or increased the actual physical distance between the participant and the technology device. Participants tended to lean away or move their chair back when frustrated with technology, which occurred in 5 video visits, while they tended to move closer or lean toward the technology when engaged with the technology or appointment, which occurred in 10 video visits. In 3 video visits, participants displayed expressive gesturing showing their engagement during the video visit with the pharmacist. Many participants were creative in adapting their environment to accommodate their device, figuring out ways to prop up their tablet on books or other objects nearby so they could use both hands to show their medication to the pharmacist or gesture freely. This adaptation occurred in 7 video visits. One participant displayed a marked physical hesitancy in interacting with the technology device. They tentatively accepted the device from the project manager and did not immediately type on the device even when prompted.

### Verbally Expresses Age or Knowledge

Participants verbally mentioned their age or knowledge of technology on average 2 (SD 2.11) times per video visit. This often came in the form of finding it difficult to keep up with technology due to one’s age (eg, “I’m too old for this,” referring to learning how to use an iPad) or aging and ailments (eg, “I have bad knees, that’s simply something that happens when you’re 70”), sometimes with humor, (eg, “wait ‘til you get to this age” in reference to questions about some chronic pains). Along the dimension of aging, participants expressed memory loss and strategies to cope with this regarding technology, for example, by writing out all passwords to remember them. Participants also expressed their limited expertise with technology (eg, regarding using an iPad, one participant said, “I don’t even use it. Not for this. I know how to make phone calls. That’s it”). Several participants explicitly stated they were technologically savvy (eg, when discussing medication supply issues due to the COVID-19 pandemic, a participant said he was getting smaller doses but stated, “I am very computer savvy, so I don’t have a problem getting on and, you know, listening to what I need”). Finally, several participants expressed reliance on younger generations when it came to learning technology (eg, stating that they learned how to use their iPad or create a passcode from their grandchild). One participant expressed pride in aging (eg, asking the pharmacist, “How many 74-year-olds do you know who can ride 25 miles on a bike?”).

### Technology Interruptions

Technology interruptions occurred in just 6 video visits once the appointment had started. In these video visits, the video either froze momentarily or was lost while the audio kept working, or there was a lag between the audio and video.

### Type of Troubleshooting

Troubleshooting the technology occurred on average 5.45 (SD 3.29) times per video visit and most often consisted of either the patient problem-solving on their own by figuring out how to flip the camera on the tablet or by asking for a pause between questions and answers with the pharmacist due to an audio lag. Project manager intervention occurred when hands-on assistance was required, such as closing a pop-up window that blocked the video or turning up the volume. Participants occasionally verbalized instructions they were following to the project manager in the room while connecting to the video visit as a form of validation that they were on the right track. There were occasional device-specific issues, such as issues connecting to the home Wi-Fi or issues accessing the camera.

### Remarks About Home, Space, or Self

Participants or the pharmacist made a remark about their home or space on average 2.75 (SD 2.12) times per video visit. This most often occurred when participants got up to retrieve medication from another room. There were also cases when there were distractions in the home, including barking dogs, an interrupting partner, or a phone ringing. In several appointments, participants using tablets (as opposed to desktops, for example) were able to give a home tour, showing the pharmacist around the home so they could see where medications were stored. Finally, in one video visit, the video heightened self-awareness*,* prompting a participant to make a joke about their “bad hair day” and asking later in the appointment if their face appeared red to the pharmacist.

### Mixed Methods Integration

The mixed methods integration showed convergence between the quantitative impression ratings and qualitative observational findings, while also clarifying how older veterans navigated telemedicine in real time (Table 6). Quantitatively, engagement was the only rating that significantly increased from technology setup to the telemedicine appointment, suggesting that participants became more involved once the visit shifted from accessing the platform to interacting with the pharmacist. Qualitative observations supported this finding. Participants leaned toward the device, became more animated, used expressive gestures, repositioned devices, and actively retrieved medications to support the medication review. In contrast, neither verbal nor nonverbal comfort nor frustration was significantly different from setup to appointment, indicating that participants’ overall comfort with technology remained relatively stable. However, qualitative data revealed that stable ratings did not mean an absence of difficulty. Participants experienced intermittent friction, including lag, frozen video, hesitation, sighing, device-specific challenges, and reliance on project manager support, while also demonstrating adaptive strategies such as propping tablets on books, moving closer to the screen, or independently troubleshooting. Together, these findings suggest that home-based pharmacist telemedicine was acceptable and feasible for this pilot sample, but its success depended on real-time support, participant adaptability, and the meaningful clinical purpose of the visit. The home environment functioned both as a source of distraction and as a clinical asset, allowing the pharmacist to observe medication storage, routines, and contextual factors that would not be visible in an office-based encounter.

**Table 6.** Joint display integrating Wilcoxon signed-rank results and qualitative findings^a^.

Quantitative finding (Wilcoxon results)	Qualitative theme	Illustrative qualitative evidence	Integrated interpretation (meta-inference)	Z Score	*P* value
Engagement increased significantly from setup to appointment: setup median 4 (IQR 4‐5); appointment median 5 (IQR 4.25‐5)	Engagement increased once the telemedicine visit began.	Participants leaned toward the device, became more animated, used expressive gestures, and actively retrieved medications or repositioned devices during the visit.	Older veterans appeared more engaged once technology use shifted from setup to a meaningful clinical interaction, suggesting that contact with the pharmacist helped overcome early hesitation.	2.03	.002
Verbally expressed comfort with technology did not significantly differ from setup to appointment: setup median 4 (IQR 2‐5); appointment median 3.15 (IQR 2‐4)	Participants expressed mixed confidence with technology.	Some participants described themselves as “too old” or limited in technology skills, whereas others described themselves as “computer savvy.”	Verbal comfort remained heterogeneous across participants, indicating varied digital confidence rather than a uniform increase in ease over the course of the visit.	1.22	.22
Verbally or vocally expressed frustration versus ease with technology did not significantly differ from setup to appointment: setup median 3 (IQR 2.25‐4); appointment median 3.20 (IQR 2.25‐3.75)	Overt frustration was limited, but friction and adaptation co-occurred.	Coders observed sighing, hesitation, leaning away from the device, lag, frozen video, and device-specific problems, along with adaptation such as propping tablets on books and moving closer when engaged.	Stable comfort and frustration ratings suggest overall acceptability, but behavioral observation shows that participants still experienced intermittent difficulty while continuing to adapt.	0.24	.81
Nonverbally expressed comfort also did not significantly differ: setup median 4 (IQR 2‐4.75); appointment median 4 (IQR 3‐5)	Overt frustration was limited, but friction and adaptation co-occurred.	Coders observed sighing, hesitation, leaning away from the device, lag, frozen video, and device-specific problems, along with adaptation such as propping tablets on books and moving closer when engaged.	Stable comfort and frustration ratings suggest overall acceptability, but behavioral observation shows that participants still experienced intermittent difficulty while continuing to adapt.	1.81	.07
Technology interruptions occurred in 6 visits; troubleshooting occurred an average of 5.45 times per visit; time to start the appointment averaged 9 minutes 4.8 seconds.	Home-based telemedicine was workable but support-dependent, and the home environment functioned as both barrier and asset.	Participants resolved some issues independently, received hands-on assistance when needed, and navigated distractions such as barking dogs or ringing phones while also retrieving medications and showing storage areas.	Telemedicine visits were feasible for most participants, but smooth participation often depended on real-time troubleshooting, environmental flexibility, and the clinical advantages of seeing medications and routines in the home.	—^b^	—

a Quantitative findings are reported as median (IQR) and Wilcoxon signed-rank test results comparing technology setup with the telemedicine appointment. Qualitative findings were derived from rapid qualitative analysis of video-recorded behavioral observations and transcripts.

b Not available.

## Discussion

### Overview of Key Findings

This descriptive pilot study provides important insights into older adults’ comfort, engagement, and challenges when using telemedicine services in their homes. Our findings highlight that, although participants were moderately engaged and expressed general ease with technology during video appointments, they also encountered various barriers that could impact the effectiveness and scalability of such services. Participants exhibited a range of nonverbal behaviors that reflected both frustration and adaptability during technology setup as well as during the video appointment. While verbal expressions of discomfort or frustration were infrequent, nonverbal cues such as sighs, increased distance from the device, and delayed responses indicated subtle discomfort for several participants. Importantly, participants demonstrated adaptability by changing their environment to facilitate engagement with technology, suggesting a willingness to overcome barriers when adequately supported.

### Implications for Telemedicine Adoption Among Older Patients

Telemedicine appointments in the home environment offer distinct advantages over in-office appointments. Appointments conducted in the individual’s home allow patients an opportunity to engage with the provider in a more comfortable environment, giving the provider more extensive insight into their cognitive and emotional state, something not as likely to be gained when in the clinic [6]. For example, telemedicine appointments allow patients to show their pharmacists how they are storing and using their medication and reduce the need for transportation, which can be particularly beneficial for older adults with multiple chronic conditions or mobility limitations [1,2]. Additionally, previous studies have also found that, even in instances in which older adults prefer in-person appointments, telemedicine access specifically is still appreciated due to its convenience, ease of access, and its prevention of exposure to infectious diseases [22]. However, despite these advantages, our findings underscore the importance of addressing technological and contextual barriers to ensure the widespread adoption of this approach.

### Technology-Related Barriers

While most participants successfully navigated technology setup and troubleshooting, some faced challenges with connectivity issues, device-specific problems, and limited technological expertise. These barriers may disproportionately affect participants with lower technological literacy, echoing concerns from prior research that older adults may have limited confidence in their ability to engage with digital platforms [8,9]. In fact, older adults have exhibited better medication adherence when they could connect with pharmacists digitally, but that adherence was significantly impacted based on understanding of device functionality [23]. Notably, participants expressed reliance on younger generations, often enlisting the help of grandchildren or other family members to learn new technologies. Addressing these challenges through targeted interventions, such as providing preappointment technology tutorials, simple written or visual instructions for joining the visit, offering real-time technical support at appointment initiation and troubleshooting guidance, assistance with audio and camera setup, workflow strategies to help patients prepare medications and identify an appropriate space before the appointment, and ensuring user-friendly interfaces, may enhance older adults’ confidence and comfort with telemedicine platforms and increase uptake. The level and type of support may need to be tailored to patients’ prior technology experience, device access, and home environment.

These findings also highlight a distinction between patient-led adaptation and staff-supported participation. Patient-led adaptations, such as repositioning devices, retrieving medications, moving closer to the screen, or asking the pharmacist to pause during lag, suggest that many older adults can actively problem-solve during telemedicine encounters. In contrast, barriers that required project manager assistance, such as closing pop-up windows, adjusting volume, resolving setup problems, or addressing connectivity issues, raise different concerns for scalability. Future telemedicine implementation should therefore not assume that successful completion of a supported pilot visit will translate directly to routine care without comparable support mechanisms.

### Contextual and Environmental Factors

The home environment introduced unique dynamics that influenced participants’ telemedicine experiences. While distractions such as barking dogs, partner interruptions, or household noises occasionally disrupted the flow of appointments, participants were able to accomplish tasks such as retrieving medications from other rooms. These distractions, which are less common in office settings, highlight the need for clinicians and telemedicine platforms to remain flexible and adaptable to home-based interruptions. Future telemedicine models may benefit from encouraging patients to identify and prepare a quiet, distraction-free space prior to the appointment, as well as incorporating strategies for managing environmental distractions when they occur. This will allow patients to avoid trips to the clinic and give their clinicians the benefit of being able to see their patients in their home environment, even allowing for explicit assessment of home safety [24].

### Addressing Perceptions of Age and Technology

Our findings also highlight the influence of age-related perceptions on participants’ engagement with telemedicine technology. Some participants voiced concerns about being “too old” to learn new technologies, reflecting self-perceptions that may hinder their willingness to engage with digital health tools. Conversely, other participants expressed pride in their technological proficiency and emphasized their ability to navigate digital platforms despite their age. These divergent perceptions underscore the need for individualized, strength-based approaches that validate participants’ experiences while offering appropriate support. Messaging that emphasizes the accessibility and ease of virtual platforms, coupled with training that acknowledges older adults’ capacity for learning and adaptation, may help mitigate negative self-perceptions and build confidence in telemedicine use.

A key strength of this study is the integration of convergent parallel design to simultaneously conduct quantitative and qualitative observational methods to capture nuanced participant experiences before and during home telemedicine appointments. The use of rapid qualitative analysis provided timely and actionable insights that align with the real-world context of telemedicine implementation. Additionally, the inclusion of impression ratings during both technology setup and the appointment itself allowed for a comprehensive assessment of changes in participants’ comfort, frustration, and engagement over time.

Several limitations warrant consideration. The sample consisted primarily of older non-Hispanic White male veterans, limiting the generalizability of findings to broader populations of older adults. Additionally, because participants were recruited through the Veterans Healthcare Administration (VHA), they may have had greater familiarity with telehealth platforms compared to older adults in non-VHA settings given the VHA’s long history of telemedicine [25,26].

Although the enrolled sample was predominantly White, this was generally consistent with the racial composition of the invited recruitment pool. Among the 316 veterans mailed invitation letters, 298 were non-Hispanic White, 1 was Hispanic White, 1 was Alaskan Native, 7 were African American, 2 were Asian, and 6 did not report race or ethnicity. Thus, the limited racial and ethnic diversity of the enrolled sample appears to reflect, in part, the composition of the invited pool rather than evidence of differential enrollment by race or ethnicity alone. However, veterans who responded to recruitment materials and completed the telepharmacy visit may have differed from nonrespondents in their willingness to participate in research, trust in VA care, interest in telehealth, access to technology, or availability of support. As a result, our findings should not be interpreted as evidence that telepharmacy is broadly acceptable to all older veterans. Rather, they describe acceptability and perceived value among a subset of older veterans who enrolled in and completed the intervention. Additionally, data collection occurred during the COVID-19 pandemic; participants’ views of telepharmacy may have been shaped by pandemic-related concerns about travel, exposure risk, and limited access to in-person care. For some veterans, COVID may have functioned as a pushing force that increased willingness to try video visits. As a result, acceptability findings may not fully reflect preferences under nonpandemic conditions. Future studies should use recruitment strategies designed to better reach veterans with limited technology access, women veterans, veterans from racial and ethnic minority groups, and those less inclined to participate in virtual care.

The presence of the project manager in participants’ homes may have affected how participants engaged with the technology and the telemedicine visit. In addition, qualitative coding and rapid analysis involved interpretive judgment, which may have been influenced by researcher training and prior assumptions about telemedicine and aging.

As a formative pilot study, this work offers feasibility insights beyond participant comfort and engagement alone. A key feasibility implication is the need to distinguish independent patient adaptation from staff-enabled visit completion, because these have different implications for scalability. First, engagement during the telemedicine appointment was generally high, and many participants demonstrated patient-led adaptation by repositioning devices, moving closer to the screen, retrieving medications, or otherwise adjusting their environment to support participation. These behaviors suggest patient capacity that may be strengthened through design, training, and clearer preparation procedures. Second, although participants generally completed the observed visits, the time required to initiate appointments, the frequency of troubleshooting, and the need for in-person assistance indicate that retention and completion in future studies may depend on reducing setup burden and strengthening preparation and planning for scalable support mechanisms. Third, training and support needs appeared substantial for some participants, particularly those with limited prior technology experience, suggesting that future telemedicine models should incorporate standardized orientation, previsit practice, simplified login procedures, and accessible technical assistance. Fourth, fidelity of intervention delivery may have been influenced by variable device types, connectivity problems, and home-environment interruptions, even though these same home-based conditions also created clinically useful opportunities for medication review and contextual assessment. Consistent with pilot and feasibility guidance, these findings can inform refinement of intervention procedures and study design before larger-scale testing. For digital health research more broadly, clearer reporting of development, support requirements, implementation context, and evaluation processes may improve transparency, reproducibility, and future uptake.

### Conclusions

This study’s primary contribution is not to establish the effectiveness of telemedicine for older adults broadly, but to provide preliminary, behaviorally grounded insight into how a small sample of older veterans navigated pharmacist-led telemedicine visits in the home environment. By combining observational ratings with qualitative analysis, the study highlights practical barriers, adaptive behaviors, and contextual features of home-based telemedicine that may not be captured through satisfaction surveys or platform-based metrics alone. While primarily non-Hispanic White, male veteran older adults demonstrated adaptability and engagement with telemedicine technology, it is important that future researchers and clinicians address barriers related to technology literacy, technical difficulties, environmental distractions, and age-related perceptions. Implementing targeted interventions that enhance technology support, simplify user interfaces, and foster confidence in digital health tools may support access and willingness to return to a telemedicine appointment for older adults managing chronic conditions.

## Supplementary material

10.2196/86944 Multimedia Appendix 1 Coding sheet.

## Abbreviations

CIRB: Central Institutional Review Board
CPP: clinical pharmacy practitioner
HIPAA: Health Insurance Portability and Accountability Act
IRB: institutional review board
VA: Veterans Affairs
VHA: Veterans Healthcare Administration
