# Supplementary material for: Acceptability and Usability of Telemedicine for Older Veterans in a Pilot Sample: Mixed Methods Study
Source: JMIR Form Res. 2026 Aug 6;10:e86944. doi: 10.2196/86944 (PMC13446794; doi:10.2196/86944)
Supplement: Supplementary file 1 [file formative-v10-e86944-s001.docx]

***Instructions:* Coders will watch the entire interaction and code for the following codes in Table 1, marking frequency or rating of each code and a short qualitative description.**

**Behavioral Observation Analysis of Acceptability and Useability of Pharmacist Telemedicine Appointments in a Veteran Sample**

Table 1

Definitions of Technology Codes

| **Code** | **Definition** |
| --- | --- |
| Asks Question or Gives Information Related to Technology | Participant had to ask a question of research assistant or pharmacist about technology or gives information about technology |
| Nonverbal Behavior Relevant to Technology Use | Note any nonverbal behavior relevant to technology use including sighs, movement towards/away from tech, etc… |
| Verbally Expresses Age/Knowledge | Participant vocally expressed that they were “too old to learn new things” or similar |
| Time Spent Troubleshooting | Time how long it takes to start appointment |
| Technology Interruptions | Note any interruptions in technology once appointment starts |
| Distracted by or Remark about Home/Space includes any comment about home/space | Participant *or* Pharmacist made comment about or was distracted by home environment/space/sounds includes comments about going into the other room to get pills etc… |

| **Impression** | **Rating Scale** |
| --- | --- |
| Verbally Expresses Comfort with Technology | 1 (expresses extreme discomfort)  2 (expresses moderate discomfort)  3 (neither expresses discomfort nor comfort)  4 (expresses moderate comfort)  5 (expresses extreme comfort) |
| Verbally/Vocally Expresses Frustration vs. Ease with Technology | 1 (expresses extreme frustration)  2 (expresses moderate frustration)  3 (neither expresses frustration nor ease)  4 (expresses moderate ease)  5 (expresses extreme ease) |
| Nonverbally Expresses Comfort with Technology | 1 (expresses extreme discomfort including distancing self from technology)  2 (expresses moderate discomfort)  3 (neither expresses discomfort nor comfort)  4 (expresses moderate comfort)  5 (expresses extreme comfort) |
| Verbal and Nonverbal Engagement with Technology | 1 (expresses extreme disengagement)  2 (expresses moderate disengagement)  3 (neither expresses disengagement nor engagement)  4 (expresses moderate engagement)  5 (expresses extreme engagement) |

Adapted from Table 1 from Williams, K., Pennathur, P., Bossen, A., & Gloeckner, A. (2016). Adapting telemonitoring technology use for older adults: a pilot study. *Research in Gerontological Nursing*, *9*(1), 17-2

**RATE PATIENT PRIOR TO PHARMACIST GETTING ON CALL:**

| **Code** | **Approximate Video**  **Start Time** | **Description of Situation/Behaviors/Dialogue** |
| --- | --- | --- |
| **Asks Question or Gives Information Related to Technology** |  |  |
|  |  |  |
|  |  |  |
|  |  |  |
|  |  |  |
|  |  |  |
|  |  |  |
|  |  |  |
|  |  |  |
|  |  |  |
| **Nonverbal Behavior Relevant to Technology Use (includes sighs, movement towards/away from tech, etc…)** |  |  |
|  |  |  |
|  |  |  |
|  |  |  |
| **Verbally Expresses Age/Knowledge** |  |  |
|  |  |  |
|  |  |  |
|  |  |  |
| **Time Spent Troubleshooting** |  |  |
|  |  |  |
|  |  |  |
|  |  |  |
|  |  |  |
| **Technology Interruptions** |  |  |
|  |  |  |
|  |  |  |
|  |  |  |
|  |  |  |
|  |  |  |
| **Distraction/Remark about Home/Space includes any comment about home/space (e.g., going into other room)** |  |  |
|  |  |  |
|  |  |  |
|  |  |  |
|  |  |  |
|  |  |  |

**RATE PATIENT FROM START OF PHARMACIST INTERACTION TO END OF CALL:**

| **Code** | **Approximate Video**  **Start Time** | **Description of Situation/Behaviors/Dialogue** |
| --- | --- | --- |
| **Asks Question or Gives Information Related to Technology** |  |  |
|  |  |  |
|  |  |  |
|  |  |  |
|  |  |  |
|  |  |  |
|  |  |  |
|  |  |  |
|  |  |  |
|  |  |  |
| **Nonverbal Behavior Relevant to Technology Use (includes sighs, movement towards/away from tech, etc…)** |  |  |
|  |  |  |
|  |  |  |
|  |  |  |
| **Verbally Expresses Age/Knowledge** |  |  |
|  |  |  |
|  |  |  |
|  |  |  |
| **Time Spent Troubleshooting** |  |  |
|  |  |  |
|  |  |  |
|  |  |  |
|  |  |  |
| **Technology Interruptions** |  |  |
|  |  |  |
|  |  |  |
|  |  |  |
|  |  |  |
|  |  |  |
| **Distraction/Remark about Home/Space includes any comment about home/space (e.g., going into other room)** |  |  |
|  |  |  |
|  |  |  |
|  |  |  |
|  |  |  |
|  |  |  |

**RATE PATIENT PRIOR TO PHARMACIST GETTING ON CALL:**

| **Impression** | **Rating** | **Comments or Exemplars** |
| --- | --- | --- |
| **Verbally Expresses Comfort with Technology** | 1 (expresses extreme discomfort)  2 (expresses moderate discomfort)  3 (neither expresses discomfort nor comfort)  4 (expresses moderate comfort)  5 (expresses extreme comfort) |  |
| **Verbally/Vocally Expresses Frustration vs. Ease with Technology (including sighs)** | 1 (expresses extreme frustration)  2 (expresses moderate frustration)  3 (neither expresses frustration nor ease)  4 (expresses moderate ease)  5 (expresses extreme ease) |  |
| **Nonverbally Expresses Comfort with Technology** | 1 (expresses extreme discomfort including distancing self from technology)  2 (expresses moderate discomfort)  3 (neither expresses discomfort nor comfort)  4 (expresses moderate comfort)  5 (expresses extreme comfort) |  |
| **Verbal and Nonverbal Engagement with Interaction via Technology** | 1 (expresses extreme disengagement)  2 (expresses moderate disengagement)  3 (neither expresses disengagement nor engagement)  4 (expresses moderate engagement)  5 (expresses extreme engagement) |  |

**RATE PATIENT FROM START OF PHARMACIST INTERACTION TO END OF CALL:**

| **Impression** | **Rating** | **Comments or Exemplars** |
| --- | --- | --- |
| **Verbally Expresses Comfort with Technology** | 1 (expresses extreme discomfort)  2 (expresses moderate discomfort)  3 (neither expresses discomfort nor comfort)  4 (expresses moderate comfort)  5 (expresses extreme comfort) |  |
| **Verbally/Vocally Expresses Frustration vs. Ease with Technology (including sighs)** | 1 (expresses extreme frustration)  2 (expresses moderate frustration)  3 (neither expresses frustration nor ease)  4 (expresses moderate ease)  5 (expresses extreme ease) |  |
| **Nonverbally Expresses Comfort with Technology** | 1 (expresses extreme discomfort including distancing self from technology)  2 (expresses moderate discomfort)  3 (neither expresses discomfort nor comfort)  4 (expresses moderate comfort)  5 (expresses extreme comfort) |  |
| **Verbal and Nonverbal Engagement with Interaction via Technology** | 1 (expresses extreme disengagement)  2 (expresses moderate disengagement)  3 (neither expresses disengagement nor engagement)  4 (expresses moderate engagement)  5 (expresses extreme engagement) |  |
